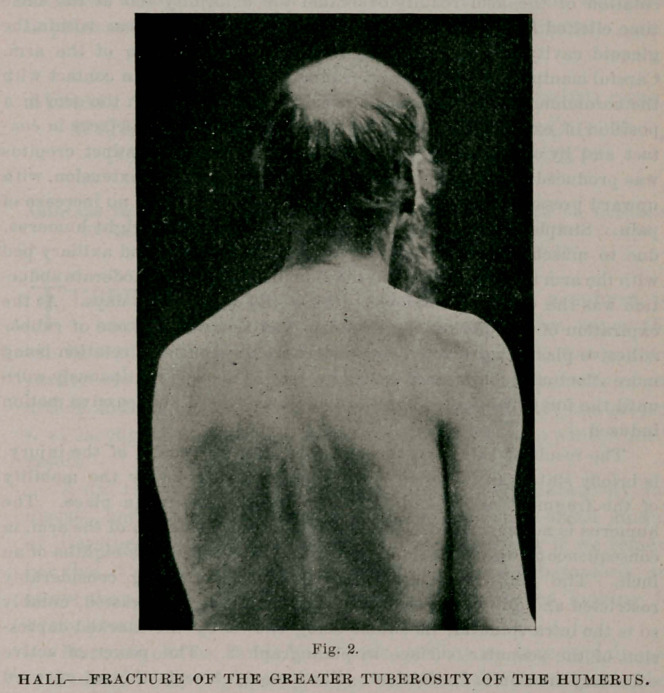# Fracture of the Greater Tuberosity of the Humerus, with the Report of a Case1Read at the twenty-eighth annual meeting of the Medical Association of Central New York, October 15, 1895.

**Published:** 1895-12

**Authors:** A. L. Hall

**Affiliations:** Fair Haven, N. Y.


### FRACTURE OF THE GREATER TUBEROSITY OF THE HUMERUS, WITH THE REPORT OF A CASE.[1]

By A. L. HALL, M. D., Fair Haven, N. Y.

FRACTURE of the greater tuberosity of the humerus is remarkable alike for its great rarity and the peculiar deformity it presents.

The simple form is extremely rare and has been met with only in a very few instances. Mayo, Hamilton, Stimson and two other writers record for themselves as having met a single case each. The fracture, however, is usually seen in conjunction with either the forward or downward variety of dislocation, and as a luxation complication it is an accident of very infrequent occurrence. Very few surgeons, of even wide experience, have ever observed a case, either in its simple or complicated form, and the systematic writers, for the most part, fail to note the history of any particular cases, from which fact it may be reasonably inferred that their individual experience has been wanting.

---

1. Read at the twenty-eighth annual meeting of the Medical Association of Central New York, October 15, 1895.

As might be expected, from our limited knowledge concerning this fracture, there exists a divergence of opinion respecting the relative importance of the causes which operate to produce it. Age, external violence and muscular action are factors of greater or less importance. Age, without doubt, is a powerful predisposing cause. In four cases of the simple fracture, the average age was fifty-five years, and in five instances, complicated with dislocation, the average age was fifty-nine years. Excluding Stimson's case, aged nineteen years, from the cases of simple fracture, the average becomes sixty-seven years. For all conditions the average age is fifty-four years. To external violence received directly upon the point of the shoulder the majority of authors attach greatest importance as a causative factor. The size and anatomical relations of the tubercle—deeply situated and highly protected by muscular coverings—appear to almost preclude the possibility of the occurrence of direct injury sufficient to produce separation. A study of the history of the recorded cases shows, by far, the greater number to have been unattended by direct outward injury, while, upon the other hand, several surgeons of note have met cases in which the separation of the tubercle was due to the powerful contraction of the spinate and small teres muscles. It is a rational conclusion, therefore, that the chief predisposing cause is advanced age, and muscular action the principal determining cause of this fracture.

The fracture is remarkable for the greatly increased width of the shoulder. Some authors assert that its breadth is nearly doubled. This is evidently a grossly exaggerated statement, as the actual increase doubtless rarely equals one inch. An abnormal flattening over the deltoid region produces an appearance of widening greater than really exists. The true increase in shoulder width is occasioned by the inward rotation of the head of the humerus, which has been released from its natural position by detachment of the tubercle and the consequent loss of opposing muscular power. The shoulder contour and the attitude of the extremity simulate somewhat closely the appearance of dislocation, for which the fracture may be mistaken. The acromion appears unusually salient, the arm is separated from the side and the elbow is thrown outward from the body.

The detached tuberosity is drawn upward and backward by muscular contraction and the head of the humerus is turned inward. Crepitus is readily elicited by rotating the humerus while direct pressure is exerted over the tuberosity, or while the head of the bone is firmly held within the grasp of the hand. The length of the arm is preserved and the thumb cannot be pressed into the glenoid cavity, as in dislocation. Deformity is easily overcome by proper manipulation, but usually returns when the arm is released. When the tough, tendinous periosteum about the

Fig. 1.

HALL—FRACTURE OF THE GREATER TUBEROSITY OF THE HUMERUS.

muscular insertion into the tubercle is untorn, there is very little displacement. The furrow betwixt the head of the bone and the separated tuberosity, of which so much has been said by writers, is more fanciful than real and, evidently, exists only in a slight degree. Some authorities state that muscular power is completely lost. This assertion is untrue, the loss of power being principally confined to active outward rotation, which is nearly or completely overcome.

Many forms of dressing have been advocated for maintaining the reduction, none of which appear to have fully met the requirements of a bad case, and it is extremely questionable if any dressing or appliance can be devised which will counteract all deformity. Most of surgeons favor the use of the axillary pad, but for what reason, unless it be from the force of habit, is not apparent. A small pad of absorbent cotton, or gauze, favors cleanliness of the parts and may prevent an annoying intertrigo, especially in hot weather; but it certainly affects nothing in retaining the parts in proper position. The various forms of splints have little or no utility in holding the fragment in place, and the claim that direct pressure may be utilised to effect this object is, to say the least, an erroneous assumption, for the tubercle is too small and too deeply placed to be efficiently controlled by pressure. The principal indication is to overcome the inward rotation of the head of the humerus. If the tendency to rotation is not present, as may be the case in incomplete separation, simply confining the arm to the side, with the forearm carried in a sling, is all that is required. In other cases, where avulsion of the tuberosity has been complete and marked inward rotation of the humerus resulted, that form of dressing should be employed which will most effectively secure the desired amount of outward rotation of the arm, thus practically restoring the tubercle to its natural site upon the head of the bone. To effect this purpose, the arm is nearly encircled with a broad strip of rubber adhesive plaster; commencing at the inner posterior surface of the arm, the plaster is carried forward over the outer surface, continued backward and attached to the back, while the arm is properly abducted. A piece of gauze is interposed betwixt the arm and the body, and the arm is secured to the side by an additional strip of plaster or by a few turns of a bandage encircling the arm and body. A sling for the forearm should not be used, as it favors inward rotation, thus defeating the object which the dressing aims to secure. The forearm should be left free and the patient should be instructed to favor outward rotation. This dressing is shown in photograph 1.

Writers generally agree that satisfactory bony union is the rule. Such a result is probable in young subjects, but in the aged, in whom the greater number of fractures occur, union, for the most part, is evidently of a fibrous character. The histories of recorded cases appear to confirm this conclusion.

Impairment of the function of the shoulder-joint apparently is the rule after this injury. The joint remains tender and painful for a long time, and when the articular surface is involved, as sometimes happens, bone spicula may complicate the case, resulting in a permanent and uncomfortable form of injury. Fibrous union is oftentimes followed by severe and lasting lameness.

Fig. 2.

HALL—FRACTURE OF THE GREATER TUBEROSITY OF THE HUMERUS.

Passive motion, if undertaken too soon, is productive of bad results and should not be instituted until union has taken place.

The following history of a recently observed case well exemplifies some of these points :

R. C., aged 64 years, laborer, strong, active and well-preserved for one of his years, while walking at a rapid pace tripped and fell, striking upon the right hand, partially breaking the fall and finally landed upon his right side. There was no bruising or other evidence of external injury, save a small superficial abrasion of the skin upon the outer surface of the lower third of the right arm. No ecchymoses were observed upon the shoulder or elsewhere at any time. Inspection revealed the characteristic widening of the shoulder and the usual flattening over the deltoid region, the arm being in a state of exaggerated adduction with the elbow projecting outward from the body. At first, dislocation was suspected, but this was speedily allayed for outward rotation of the arm readily overcame the deformity and at the same time elicited faint crepitus. The head of the humerus was within the glenoid cavity and moved freely in response to rotation of the arm. Careful manipulation disclosed a fragment of bone lying in contact with the acromion. Pressure exerted over the fragment, with the arm in a position of extreme abduction, brought the separated surfaces in contact and by outward movement of the humerus very distinct crepitus was produced. There was no shortening of the arm and extension, with upward pressure from the point of the elbow, provoked no increase of pain. Simple fracture of the greater tuberosity of the right humerus, due to muscular action, was diagnosticated. A sling and axiliary pad with the arm firmly bandaged to the side in a position of moderate abduction was the dressing employed during the first twelve days. At the expiration of this period the dressing was removed and one of rubber adhesive plaster substituted with better results ; outward rotation being more effectually maintained under its use. This was continuously worn until the forty-fifth day, when the arm was released and passive motion induced.

The result of this case, three months after the receipt of the injury, is briefly stated as follows : fibrous union, as shown by the mobility of the fragment and the presence of crepitus, has taken place. The humerus is not shortened. There is some inward rotation of the arm, in consequence of which the shoulder width is increased three-eighths of an inch. The joint function is impaired, motion being considerably restricted and quite painful. The scapular muscles are wasted, notably so is the infra-spinatus, its extent being shown by the marked depression of the scapular surface in photograph 2. The power of active voluntary motion is diminished. Particularly true is this of outward rotation, which is feebly performed. Much improvement of the present condition will unquestionably follow and, upon the whole, the probable result will be better in this instance than is usual with cases occurring at an advanced period of life.[1]

The deductions to be derived from what has been presented concerning fracture of the greater tuberosity of the humerus may be briefly summarised as follows :

1. It is an occurrence of great rarity.

---

1. The photographic views presented were taken from this case three months after the receipt of the injury.

2. The chief predisposing cause is advanced age.

3. The most frequent determining cause is muscular action.

4. There is but one principle of treatment involved—that of proper humeral abduction.

5. Fibrous union, considerable permanent widening of the shoulder, diminished power of outward arm rotation with some impairment of joint function is the rule after this fracture.

## Figures and Tables

**Fig. 1. f1:**
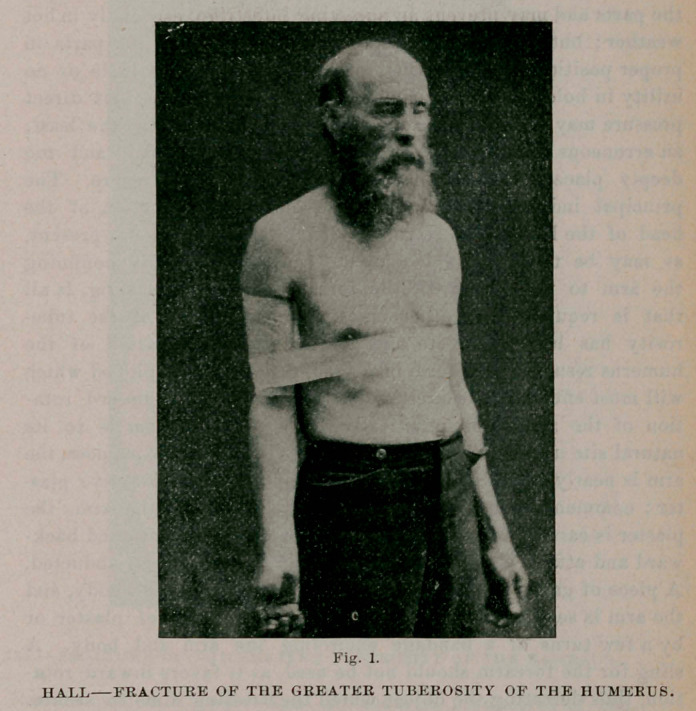


**Fig. 2. f2:**